# Can seizure therapies and noninvasive brain stimulations prevent suicidality? A systematic review

**DOI:** 10.1002/brb3.2144

**Published:** 2021-04-10

**Authors:** Yiming Chen, Charline Magnin, Jérome Brunelin, Edouard Leaune, Yiru Fang, Emmanuel Poulet

**Affiliations:** ^1^ Shanghai Mental Health Center Shanghai Jiao Tong University School of Medicine Shanghai China; ^2^ Department of Emergency Psychiatry Edouard Herriot Hospital Hospices Civils de Lyon Lyon France; ^3^ INSERM U1028, CNRS UMR5292 Lyon Neuroscience Research Center, PSYR2 Team University of Lyon, CH Le Vinatier Lyon France; ^4^ CAS Center for Excellence in Brain Science and Intelligence Technology Shanghai China; ^5^ Shanghai Key Laboratory of Psychotic disorders Shanghai China

**Keywords:** cranial electrostimulation, electroconvulsive therapy, repetitive transcranial magnetic stimulation, suicide, transcranial direct current stimulation

## Abstract

**Background:**

Suicide is a major public health issue and the majority of those who attempt suicide suffer from mental disorders. Beyond psychopharmacotherapy, seizure therapies and noninvasive brain stimulation interventions have been used to treat such patients. However, the effect of these nonpharmacological treatments on the suicidal ideation and incidence of suicidality remains unclear. Here, we aimed to provide an update on the effects of seizure therapies and noninvasive brain stimulation on suicidality.

**Methods:**

We conducted a systematic review of the literature in the PubMed, EMBASE, Cochrane Central Register of Controlled Trials, Elsevier ScienceDirect, and Wiley Online Library databases using the MeSH terms “Electroconvulsive Therapy”, “Magnetic Seizure Stimulation”, “repetitive Transcranial Magnetic Stimulation”, “transcranial Direct Current Stimulation”, “Cranial Electrostimulation” and “suicide”. We included studies using seizure therapies and noninvasive brain stimulation as a main intervention that evaluated suicidality, regardless of diagnosis.

**Results:**

Among 1,019 records screened, 26 studies met the inclusion criteria using either electroconvulsive therapy (*n* = 14), magnetic seizure therapy (*n* = 2), repetitive transcranial magnetic stimulation (*n* = 9), or transcranial direct current stimulation (*n* = 1). We observed that studies reported significant results, suggesting these techniques can be effective on the suicidal dimension of mental health pathologies, but a general statement regarding their efficacy is premature due to limitations.

**Conclusions:**

Future enquiry is necessary to address methodological limitations and evaluate the long‐term efficacy of these methods both alone and in combination with pharmacotherapy and/or psychotherapy.

## Graphical abstract

We provided an update of current evidence on the effects of seizure therapies (electroconvulsive therapy and magnetic seizure therapy) and noninvasive brain stimulations (repetitive transcranial magnetic stimulation, transcranial direct current stimulation, and transcranial electrostimulation) on suicidality. The antisuicidal effect of ECT could be supported, but magnetic seizure therapy, repetitive transcranial magnetic stimulation, transcranial direct current stimulation, and transcranial electrostimulation were not consistently recommended for treating suicidality. Long‐term antisuicidal effect of seizure therapies and noninvasive brain stimulations should be further investigated both alone and in combination with pharmacotherapy or psychotherapy.

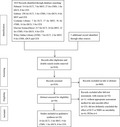

## 1 INTRODUCTION

According to the World Health Organization, suicide leads to approximately 800,000 deaths every year (WHO, 2019). The worldwide number of suicide attempts is even higher with a rate of 16 per 100,000 individuals, and based on data provided by the European Psychiatric Association, the rates of attempted suicides rate among males and females were 18 and 11 per 100,000, respectively (Plans et al., 2018). Every suicide is a tragedy for their families, communities, and entire countries for a long period. Suicides may occur throughout the lifespan and were the second leading cause of death among 15‐ to 29‐year‐olds in 2016. Suicide not only occurs in high‐income countries, but it is also a global phenomenon in all regions of the world, with over 79% of the completed suicides occurring in low‐ and middle‐income countries (WHO, 2019). Mental disorders, especially depression, are frequently found in the psychological reports of suicides in Asian as well as Western countries (Cheng et al., 2000; Phillips et al., 2002).

Given the high suicide risk of patients diagnosed with mental illnesses, it is important to track whether specific treatment strategies have the potential to rapidly and effectively reduce suicide risk. The common treatment approaches—such as pharmacotherapy and psychotherapy—have over years emerged as essential interventions reducing suicidality. However, lithium and ketamine, while effective, have various limitations such as drowsiness, dizziness, poor coordination, toxicity, and risk for dependency (Andrade, 2017; Zalsman et al., 2016). Regarding psychotherapy, a recent meta‐analysis concluded that dialectical behavior therapy reduced self‐directed violence and resulted in less psychiatric crisis interventions; however, it did not reduce suicidal ideation (DeCou et al., 2018). Therefore, to decrease suicidal ideation and prevent suicide attempts other alternative or adjunct methods are warranted. Among them, seizure therapies and noninvasive brain stimulation methods have been proposed as possible interventional tools.

Seizure therapies such as electroconvulsive therapy (ECT), magnetic seizure therapy (MST), and noninvasive brain stimulation (NIBS) methods such as repetitive transcranial magnetic stimulation (rTMS), transcranial direct current stimulation (tDCS), and cranial electrostimulation (CES) are under investigation (Desmyter et al., 2011). They may alleviate symptoms in several psychiatric conditions depending on the targeted psychiatric condition and application (Lefaucheur et al., 2014, 2017; Milev et al., 2016; Rosa & Lisanby, 2011; UK ECT Review Group, 2003). However, their efficacy on suicidality is still unclear. The aim of the present systematic review was to evaluate the effects of these applications on suicidality, that is, suicidal ideation, suicide attempt, and/or completed suicide.

## 2 METHODS

### 2.1 Search strategy and selection criteria

We performed a systematic review of the literature according to PRISMA guidelines (Liberati et al., 2009) including full‐length original articles published in English and peer‐reviewed journals until 1 June 2019. This systematic literature search was conducted in the PubMed, Cochrane Central Register of Controlled Trials, Elsevier ScienceDirect, and Wiley Online Library (STM) databases using a term list including words relative to ECT, MST, rTMS, tDCS, CES, and suicidality. We used words with combination of thesaurus [MeSH Terms] related to medical subject heading description, and [Other Term] related to keywords used in studies: (1) “Electroconvulsive Therapy” AND “suicide”; (2) “Magnetic seizure stimulation” AND “suicide”; (3) “repetitive Transcranial Magnetic Stimulation” AND “suicide”; (4) “Transcranial Direct Current Stimulation” AND “suicide”; (5) “Cranial Electrostimulation” AND “suicide”.

Two independent investigators (YC and CM) screened the results according to the eligibility criteria, first on titles and abstracts and then on full‐text articles. Eligibility criteria were as follows: (1) interventions designed for patients with a mental health disorder with available suicidality assessment as a main or secondary outcome; (2) seizure therapies (ECT and/or MST) and/or NIBS methods (rTMS and/or tDCS and/or CES) specified as the main interventions in acute or continuative phase; and (3) quality of the design corresponding to level 1, 2, or 3 on the Sackett scale. Duplicate articles and double search results were identified and removed throughout the search process.

### 2.2 Data extraction and study quality assessment

Methods and results were extracted for each included article. We paid special attention to participant characteristics (sample size, diagnosis, treatment), study design, statistical analysis, and reported findings (immediately after treatment and long‐term); next, we analyzed similarities and differences between articles with the aim of verifying which parameters were effective and for which mental disease.

Each study design was evaluated according to the modified Sackett Scale which is based on physiotherapy evidence database (PEDro) scores for its level of evidence grade. The Sackett Scale involves 5 levels of evidence: level 1—high‐quality randomized controlled trial (RCTs) (PEDro ≥6), divided into level 1a and 1b, based on the number of RCTs supporting the evidence; level 2—RCTs (PEDro score <6), cohort studies, and prospective controlled trials; level 3—case controls such as retrospective studies comparing conditions; levels 4 and 5—case reports, case series, uncontrolled pre–post‐tests, and observational studies.

In addition, we referred to the grading of recommendations, assessment, development, and evaluations (GRADE) method to rate the quality of evidence. GRADE presents four grades as levels of evidence: high, moderate, low, and very low, considering different factors such as bias related to imprecision, inconsistency, publication, and indirectness of the study (Guyatt et al., 2011).

## 3 RESULTS

**FIGURE 1:**
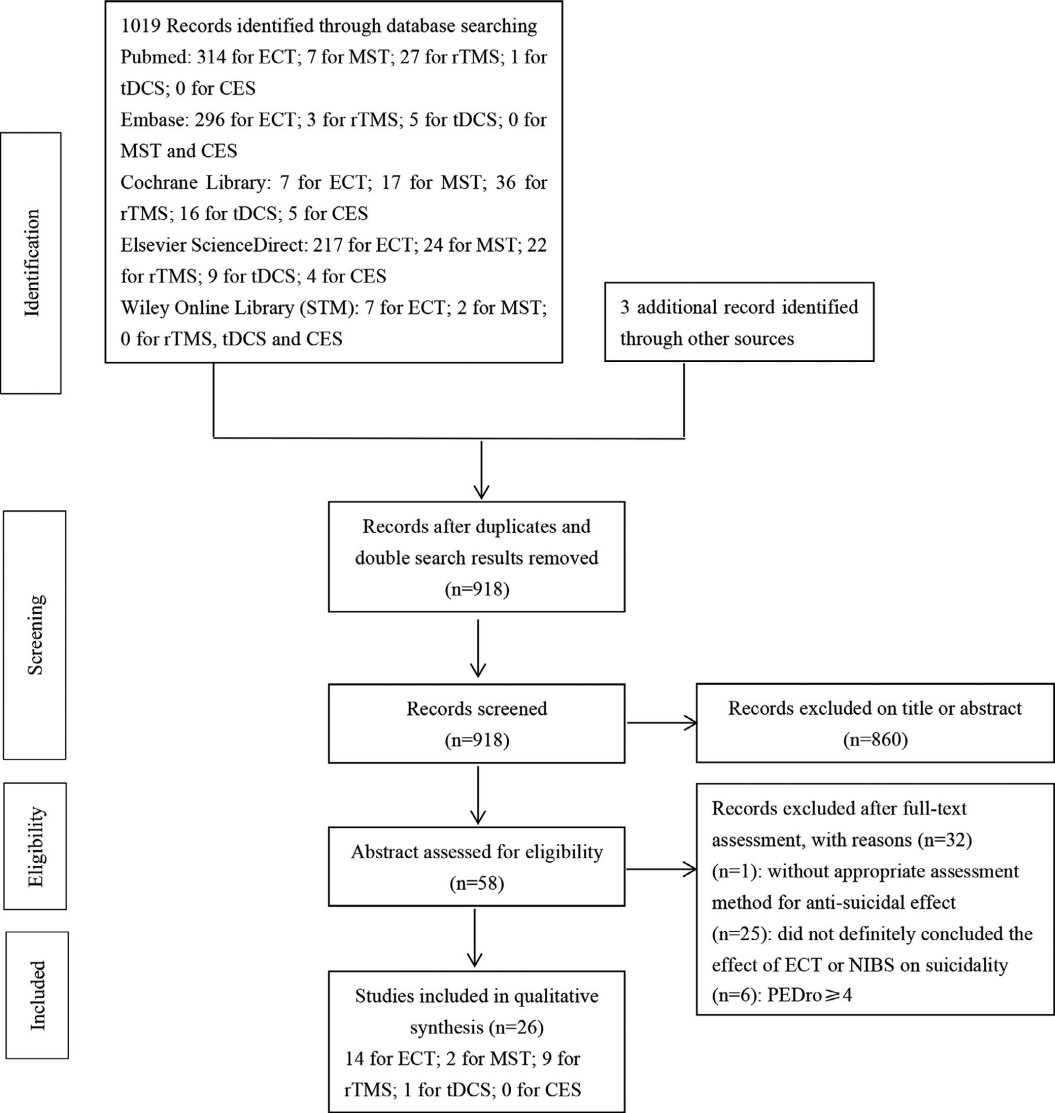
Flow diagram of study selection and inclusion—clean version

The literature search retrieved 1,019 articles. After duplicates and double search results were removed and titles and abstracts were assessed, 58 articles were selected for further evaluation. Among them, 26 studies met all inclusion criteria following full‐text evaluation and were systematically analyzed. Due to the search results, George et al. (2014) included suicidal patients with mild traumatic brain injury was also accepted in our review. Berlim et al. (2014), Desmyter et al. (2016), and Hadley et al. (2011), were accepted as additional record identified through other sources by reading references of Weissman et al. (2018). Figure 1 shows the results of the search and selection process.

### 3.1 Characteristics of studies

Fourteen ECT studies, two MST studies, nine rTMS studies, and one tDCS study were included in the present review. Among them, one nonblinded randomized trial involved therapeutic applications of both ECT and rTMS (Keshtkar et al., 2011) for major depressive disorder (MDD). There were other two nonblinded randomized trials which applied ECT for MDD (Kellner et al., 2005; Nordenskjöld et al., 2013), three prospective controlled studies for bipolar disorder (Ciapparelli et al., 2001), for depressed elderly individuals (Veltman et al., 2018), and for depressive symptoms (Avery & Winokur, 1978). Eight retrospective studies of ECT for different kinds of mental illnesses were included (Ahmadi et al., 2015; Black et al., 1989; Brådvik & Berglund, 2006; Hunt et al., 2011; Liang et al., 2017; Munk‐Olsen et al., 2007; Patel et al., 2006; Popiolek et al., 2018). Two prospective MST studies (Sun et al., 2016, 2018) were designed for patients with treatment‐resistant depression (TRD). Five RCTs (Baeken et al., 2019; Desmyter et al., 2014, 2016; George et al., 2014; Weissman et al., 2018) and two prospective studies, Berlim et al. (2014) and Hadley et al. (2011) and using rTMS and deep transcranial magnetic stimulation (dTMS), respectively, and other two prospective studies (Croarkin et al., 2018; Wall et al., 2011) designed specifically for adolescents with suicidal ideation, were included. Only one RCT using tDCS was designed for patients with acute depressive episodes (Brunoni, Júnior, et al., 2013; Brunoni, Valiengo, et al., 2013).

The methodological quality of the six RCTs was high, five with a Sackett Scale level 1a (Baeken et al., 2019; Brunoni, Júnior, et al., 2013; Brunoni, Valiengo, et al., 2013; Desmyter et al., 2014, 2016; George et al., 2014), and the other one was rated as 1b (Weissman et al., 2018). However, Weissman et al. (2018) pooled data from two other published RCTs (Blumberger et al., 2011, 2016) which were all designed to compare the depressive remission rate by rTMS in patients with TRD and were guided by their own research team to measure suicidal ideations. Five ECT studies (Avery & Winokur, 1978; Ciapparelli et al., 2001; Kellner et al., 2005; Nordenskjöld et al., 2013; Veltman et al., 2018), two MST studies (Sun et al., 2016, 2018), and three rTMS studies (Berlim et al., 2014; Hadley et al., 2011; Wall et al., 2011) rated level 2 were designed as nonblinded randomized trials or prospective studies. The eight remaining ECT researches (Ahmadi et al., 2015; Black et al., 1989; Brådvik & Berglund, 2006; Hunt et al., 2011; Liang et al., 2017; Munk‐Olsen et al., 2007; Patel et al., 2006; Popiolek et al., 2018) were all rated level 3. Overall, the level of evidence can be generally considered low, with only six studies out of twenty‐six having an evidence level of 1.

### 3.2 Participant characteristics

This review included twenty‐six studies involving 3, 589 patients diagnosed with different mental disorders: affective disorders (unipolar depression, bipolar disorder, or anxiety disorders) (*n* < 3, 710), post‐traumatic stress disorder (PTSD) (*n* = 92), personality disorders (*n* = 2), mix mania (*n* = 41), schizophrenia (*n* = 50), schizoaffective disorders (*n* > 60), and other nonaffective psychosis (*n* = 9) or other disorders (*n* = 48) referred to suicide. As for Avery & Winokur (1978) did not provide a clear description of participant diagnosis, we could not accurately calculate the sample of affective disorders and schizoaffective disorders. Heterogeneity was present among the participant demographics (Table 1).

**TABLE 1:** Summary of the Participant's characteristics

Study	Sample (Stimulation group)	Diagnosis and diagnosis instruments	Male	Female	Mean age (*SD*)		Treatment	Suicide characteristics compared	Medication
Kellner et al. (2005)	444	Unipolar MDD, DSM‐IV, 24‐HDRS≥21	141	303	55.6 ± 16.8		Bifrontal ECT; Bifrontal ECT+pharmacotherapy	Suicidal ideation of 24‐HDRS	Nortriptyline+lithium
Brådvik and Berglund (2006)	195	Melancholia, DSM‐IV and a multiaxial diagnostic schedule at discharge	/	/	/		Pharmacotherapy; ECT; ECT+continuation treatment with antidepressants	Suicidal gestures, occurrence of suicide attempt	Antidepressant pharmacotherapy
Liang et al. (2017)	487	Unipolar disorder or bipolar disorder, ICD−9‐CM	174	313	38.5 ± 14.2 (ECT group)		ECT	Completed suicide	Not specified
Ahmadi et al. (2015)	92	Both MDD and PTSD, DSM‐IV and ICD	78	14	52 ± 12 (MDD and PTSD with ECT)		Bifrontal ECT	Completed suicide	Antidepressant monotherapy
Keshtkar et al., 2011	40	MDD, DSM‐IV	21	52	35.6 ± 8.1 (ECT group)		Bilateral ECT, active rTMS	Suicide ideation of BDI and HDRS	Not specified
Patel et al. (2006)	30	Bipolar disorder, Major depression, Schizoaffective disorder, DSM‐IV	19	11	32.9 ± 11.3 (Mental ill, ECT group)		Bilateral ECT	Suicide ideation of 24‐BPRS	Psychotropic medications
					37.7 ± 9.1 (Mental ill substance abuse, ECT group)				
Ciapparelli et al. (2001)	64	Mixed mania and bipolar depression, DSM‐IV	40	24	38.0 ± 11.8 (mixed mania)		Bilateral ECT	Suicide ideation of MADRS	Lithium, anticonvulsants, TCAs, SSRIs, neuroleptics
					40.5 ± 14.3 (bipolar depression)				
Veltman et al. (2018)	89	Unipolar MDD, DSM‐IV, MINI	30	59	73.4 ± 9.8		Bilateral, unilateral ECT	Suicidal ideation of MADRS	Antipsychotics were tapered off before starting ECT but were allowed if clinically indicated
Hunt et al. (2011)	71	Affective disorder, schizophrenia, alcohol dependence, drug dependence, personality disorder, anxiety disorders, etc, /	/	/	54 (group received ECT at the time of death)		ECT	Completed suicide	Not specified
Munk‐Olsen et al. (2007)	783	Schizophrenia, schizoaffective disorders, bipolar disorders, unipolar depressive disorders, other nonaffective psychosis, other disorders, ICD‐8, and ICD‐10	/	/	/		Bilateral, unilateral ECT	Completed suicide	Not specified
Popiolek et al. (2018)	109	Bipolar depression, ICD‐10	/	/	/		cECT	Suicide attempt or completed suicide	Not specified
Black et al. (1989)	372	Primary unipolar, secondary unipolar, bipolar depressives, DSM‐III	/	/	/		ECT, an adequate trial of antidepressants, an inadequate trial of antidepressants, a group receiving neither ECT nor antidepressants	Completed suicide	Antidepressant monotherapy
Brunoni, Júnior, et al. (2013) and Brunoni, Valiengo et al. (2013)	28	Major depression (single episode, recurrent, or bipolar), MINI‐PLUS, MADRS, CGI	28	28	52 ± 17 (ECT Plus Pharmacotherapy)		cECT	Suspected suicide and suicide attempt	Pharmacotherapy alone
					62 ± 13 (Pharmacotherapy alone)				
Avery and Winokur (1978)	257	Manic‐depressive, depressed; manic‐depressive, circular or mixed; psychotic depressive reaction; involutional melancholia; depressive neurosis; and schizoaffective, DSM‐I or DSM‐II	/	/	/		ECT, antidepressant therapy, and neither ECT nor antidepressants	Suicidal ideation, suicide attempt and completed suicide	Imipramine, amitriptyline, desipramine, or nortriptyline
Sun et al. (2016)	27	TRD, DSM‐IV	12	15	46.0 ± 15.3		MST	Suicidal ideation of SSI	Benzodiazepine medication
Sun et al. (2018)	23	TRD, DSM‐IV	11	12	45.0 ± 12.2		MST	Suicidal ideation of SSI	Antidepressant, antipsychotic, benzodiazepine, lithium, pregabalin
Weissman et al. (2018)	156	TRD, DSM‐IV	59	97	Blumberger et al (2012)	Blumberger et al. (2016)	Bilateral rTMS; L‐DLPFC‐rTMS and a sham control condition	Suicidal ideation of HDRS	Not specified
					58.0 ± 12.5 (Bilateral)	46.4 ± 12.5 (Bilateral)			
					48.9 ± 13.4 (Unilateral)	46.5 ± 14.1 (Unilateral)			
					45.8 ± 13.4 (Sham)	48.1 ± 12.0 (Sham)			
Wall et al. (2011)	8	MDD, DSM‐IV, CDRS‐R ≥ 40	1	7	16.5		L‐DLPFC‐rTMS	Suicide ideation of the Suicide Severity Rating Scale	SSRI
Hadley et al. (2011)	19	Depressive episode, BDI≥12	8	11	48 ± 16		L‐DLPFC‐rTMS	Suicidal ideation of SSI	Not specified
Desmyter et al. (2016)	50	TRD, MINI, 17‐HDRS≥14	15	35	41.9 ± 11.77		L‐DLPFC iTBS‐sham, sham‐L‐DLPFC iTBS	Suicidal ideation of SSI	No medication
George et al. (2014)	41	Unipolar or bipolar depression, DSM‐IV, SSI≥12, 3 of HDRS≥3	35	6	42.5 ± 15.7		L‐DLPFC‐rTMS, sham stimulation	Suicidal ideation of SSI	Not specified
Desmyter et al. (2014)	12	TRD, MINI	5	7	44.91 ± 10.8		L‐DLPFC iTBS, sham stimulation	Suicidal ideation of SSI	No medication
Baeken et al. (2019)	45	MDD, MINI	12	33	44 ± 19		L‐DLPFC iTBS‐sham, sham‐L‐DLPFC iTBS	Suicidal ideation of SSI	No medication
Croarkin et al. (2018)	19	TRD, ATHF	6	13	16.0 ± 1.29		L‐DLPFC‐rTMS	Suicidal ideation of C‐SSRS and CDRS‐R	SSRI or SNRI
Berlim et al. (2014)	17	MDD, MINI, 21‐HAMD≥18	13	4	47.12 ± 13.26		L‐DLPFC DTMS	Suicidal ideation of SSI	Not specified
Brunoni, Júnior, et al. (2013) and Brunoni, Valiengo, et al. (2013)	120	Acute depressive episode, MINI	38	82	46.4 ± 14 (Placebo)		Sertraline‐only, tDCS‐only, combined‐treatment, placebo	Suicide ideation of MADRS	Sertraline, benzodiazepine

Abbreviations: 24‐BPRS, 24‐item Brief Psychiatric Rating Scale; ATHF, Antidepressant Treatment History Form; BDI, Beck Depression Inventory; CDRS‐R, Children's Depression Rating Scale‐Revised; cECT, continuation ECT; DSM‐IV, Diagnostic and Statistical Manual of Mental Disorders, IV Edition;DTMS, deep transcranial magnetic stimulation; HDRS, Hamilton depression scale; ICD, The International Statistical Classification of Diseases and Related Health Problems; iTBS, intermittent theta burst stimulation; L‐DLPFC, Left dorsolateral prefrontal cortex; MADRS, Montgomery–Asberg Depression Rating Scale; MINI, The Mini International Neuropsychiatric Interview; MMSE, Mini‐Mental State Examination; PTSD, post‐traumatic stress disorder; rTMS, repetitive transcranial magnetic stimulation; SNRIs, Serotonin and Noradrenaline Reuptake Inhibitors; SSI, Beck Scale of Suicidal Ideation; SSI‐CV, Beck Scale for Suicide Ideation‐Chinese Version; SSRIs, Selective Serotonin Reuptake Inhibitors; TRD, treatment‐resistant depression.

### 3.3 Intervention

#### 3.3.1 Diagnostic tools

All the studies but two studies (Hadley et al., 2011; Hunt et al., 2011) used a diagnostic tool to select participants at baseline such as the Diagnostic and Statistical Manual of Mental Disorders (DSM) (*n* = 14), the International Statistical Classification of Diseases and Related Health Problems (ICD) (*n* = 4), the Mini International Neuropsychiatric Interview (MINI) (*n* = 7), or Antidepressant Treatment History Form (ATHF) (*n* = 3). Only twelve studies provided clear exclusion criteria at baseline.

#### 3.3.2 Stimulation protocol

Only half of the ECT studies described the treatment parameters. The parameters included features such as current, duration, frequency, and pulse width. Keshtkar et al. (2011) and Ciapparelli et al. (2001) used the MECTA Corporation model device (MECTA Corp, Lake Oswego, Ore) while Veltman et al., 2018), Ahmadi et al. (2015), and Patel et al. (2006) applied the Thymatron System IV (Somatics, Inc, Lake Bluff, Ill). Nordenskjöld et al. (2013) used the both. The only relevant variation concerning the frequency of sessions ranged between 1 session per month and 3 sessions per week. Technical characteristics of ECT are presented in Table 2.

**TABLE 2:** Summary of ECT study characteristics

Study	Study design	Stimulus dose administration	Number of sessions	Frequency of treatment		Analysis	Primary Outcome	Results/Authors' conclusions	Evidence (PEDro)
Kellner et al. (2005)	A nonblinded randomized trial	a Thymatron DGx device	Treatment group: 13 sessions	ECT group	PT group	Baseline, 1st w, 2nd w	24‐HDRS	Expressed suicidal intent in depressed patients could be rapidly relieved with ECT.	Level 1b
			Control group: 3 sessions+PT (nortriptyline+lithium)	Acute phase	Acute phase				
				1st w: 3 sessions	1st w: 3 sessions				
				Continuation ECT	Continuation PT				
				2nd−5th w: 1 session/w	nortriptyline+lithium				
				6th−13th w: 1 sessions/2 w					
				The following 2 m: 1 session/m					
Brådvik and Berglund (2006)	A retrospective study	Unknown	ECT group: at least 6 sessions	ECT group: 3 sessions/w		After 28 years and 32 years	/	Occurrence of suicidal attempt: ECT+continuation treatment with ATD<ECT<ATD. Seriousness of suicide attempt: ECT>ATD.	Level 3
			ECT+continuation treatment with ATD group: 6 sessions	ECT+continuation treatment with antidepressant group: 3 sessions/w during 2 weeks+continuation with ATD					
Liang et al. (2017)	A nationwide retrospective cohort study	Unknown	Unknown	Unknown		From 1 January 2000 to 31 December 2013	/	Suicide events: ECT group<Non‐ECT group	Level 3
								Cumulative risk of suicide: ECT group<Non‐ECT group (All patients with AD). Cumulative risk of suicide: ECT group<Non‐ECT group (Patients with UD). Cumulative risk of suicide: ECT group=Non‐ECT group (Patients with BD).	
Ahmadi et al. (2015)	A retrospective nested matched case–control study	A Thymatron IV ECT device	6 ± 1 sessions	3 sessions/week		Baseline, 12th week	CGI‐S, CGI‐C	The suicide rate: 2.2% in ECT group and 5.9% in ATD group	Level 3
								The relative‐risk of suicidality was 64% less in MDD and PTSD with ECT treatment compared to those without ECT.	
Keshtkar et al. (2011)	A nonblinded randomized trial	ECT group: (MECTA Corporation model device)	10 sessions (ECT or rTMS)	ECT group	**rTMS group**	Preintervention and postintervention	HDRS, BDI	The decrease in the score of suicide in the short term: ECT>rTMS.	Level 1b
		rTMS group: 90%RMT, Frequency: 10 Hz for either 2s or 6s, Train: 20s		3 sessions/w	1 session/d				
Patel et al. (2006)	A retrospective study	A Thymatron System IV	5–10 sessions	3 sessions/w		Pre‐ECT and post‐ECT	24‐BPRS	Efficacy of suicide for depressive patients: ECT>control group.	Level 3
Ciapparelli et al. (2001)	A prospective controlled study	MECTA Corporation model device	MM: 7.2 ± 1.7 sessions; BD: 7.3 ± 1.6 sessions	2 sessions/w		T0, T1, T2	MADRS, BPRS, CGI‐S	The decrease in suicidality: MM>BD from T0 to T2.	Level 2
Veltman et al. (2018)	A prospective controlled study	A Thymatron System IV	4 sessions	2 sessions/w		Baseline, 1st w, 2nd w	MADRS	Suicidality showed significant improvement in w 1 as compared with baseline.	Level 2
Hunt et al. (2011)	A retrospective study	Unknown	Unknown	Unknown		In 2011	/	The fall in the use of ECT has not affected suicide rates in patients receiving this treatment.	Level 3
Munk‐Olsen et al. (2007)	A register‐based cohort study	Unknown	Unknown	Unknown		In 2007	/	Patients who had received ECT had a slightly higher suicide rate, especially within the first 7 days after the last ECT treatment.	Level 3
Popiolek et al. (2018)	A retrospective study	Unknown	5.8 ± 5.3 sessions	Unknown		From January 2011 to December 2014	/	Patients treated with cECT had a similar risk of suicide attempt or completed suicide to those who did not receive cECT.	Level 3
Black et al. (2016)	A retrospective study	Unknown	Unknown	Unknown		From January 1970 to December 1981		From a short‐term follow‐up of depressives that ECT received in the hospital has minimal influence on subsequent mortality, including suicide.	Level 3
Brunoni, et al. (2013)	A multicenter nonblinded randomized trial	The Mecta Spectrum 5000Q device and a Thymatron System IV	29 sessions	Weekly cECT for 6 weeks and thereafter every 2 weeks for 46 additional weeks, a total of 29 ECTs for the full year.		The patients were recruited between 10 January 2008 and 22 March 2012. Follow‐up was completed on 29 May 2012.		One suspected suicide and 3 suicide attempts by intoxication occurred, all in the PT‐alone group.	Level 2
Avery and Winokur (1978)	A prospective study	/	Unknown	Unknown		From 1959 to 1960 and from 1967 to 1968		Suicide attempts were seen significantly less frequently in the ECT groups than in the ATD group or the "adequate" ATD subgroup. Fewer suicide attempts occurred in the ECT group compared to the ATD group among both in those who had attempted suicide prior to admission (0% versus 10%) and in those who had not (1.1% versus 3.6%).	Level 2

Abbreviations: AD, Affective disorders; ATD, Antidepressant; BD, Bipolar disorder; BDI, Beck Depression Inventory; 24‐BPRS, 24‐item Brief Psychiatric Rating Scale; CGI‐C, Clinician Global Impressions‐Change scale; CGI‐S, Clinician‐rated global illness severity; ECT, Electroconvulsive Therapy; HDRS, Hamilton depression scale; MADRS, Montgomery–Asberg Depression Rating Scale; MM, mixed mania; RCT, randomized controlled trial; T0, the day before starting ECT; T1, 48 hr after completion of the 3rd session; T2, a week after the last session; PEDro, the Physiotherapy Evidence Database tool; PT, pharmacotherapy; UD, unipolar disorder.

MST intervention of these two studies was delivered with a MagPro MST machine (MagVenture) and a twin coil symmetrically placed over the frontal cortex with the maximal electric field over the Fz electrode position according to the international 10–20 system (DaSilva et al., 2011). Details are provided in Table 3.

**TABLE 3:** Summary of MST study characteristics

Study	Study design	Stimulus dose administration	Number of sessions	Frequency of treatment	Analysis	Primary Outcome	Results/Authors' conclusions	Evidence (PEDro)
Sun et al. (2016)	A prospective controlled study	MagPro MST	24 sessions	/	Baseline, after 24 sessions	HDRS‐24, SSI, ATHF	Pre–post‐treatment mean difference of SSI is 4.8 ± 6.7.	Level 2
Sun et al. (2018)	A prospective controlled study	MagPro MST	24 sessions	/	Baseline, after 24 sessions	HDRS‐24, SSI, ATHF	44.4% of patients experienced resolution of suicidal ideation.	Level 2

Abbreviations: ATHF, Antidepressant Treatment History Form; HDRS, Hamilton depression scale; MST, magnetic seizure stimulation; SSI, Beck Scale of Suicidal Ideation.

Intensity (expressed as % of the resting motor threshold (RMT)), frequency of stimulation, frequency of sessions, train duration and intertrain intervals, type of coil, and coil location are important parameters that influence the effects of rTMS. A large variability of these parameters was observed between studies, while a large majority of studies used high‐frequency (10 Hz) rTMS with a figure 8 coil over the left dorsolateral prefrontal cortex (DLPFC). Desmyter et al. (2016) and Desmyter et al. (2014) used accelerated intermittent theta burst stimulation (iTBS), while Baeken et al. (2019) used placebo‐accelerated iTBS. Blumberger et al. (2016) used bilateral stimulation (high‐frequency rTMS over the left DLPFC, low‐frequency rTMS – 1Hz over the right DLPFC), and Berlim et al. (2014) used deep TMS at 20Hz delivered with a “H1” coil over the left DLPFC. Intensity of stimulation varied across studies from 100% to 120% RMT. The total number of sessions varied from 7 to 30, while the frequency of sessions was 5 sessions per week at the lowest and 5 sessions per day at the highest. Details of rTMS parameters are provided in Table 4.

**TABLE 4:** Summary of rTMS study characteristics

Study	Study design	Stimulus dose administration				Number of sessions	Frequency of treatment	Analysis	Primary outcome	Results/Authors' conclusions	Evidence (PEDro)
Weissman et al. (2018)	RCT	Blumberger et al., 2011)		Blumberger et al. (2016)		15 sessions	5 sessions/w	Baseline, 1st w, 2nd w, 3rd w, 4th w, 5th w, 6th w	24‐HDRS	Suicidal ideation: Bilateral>Sham	Level 1b
		Left unilateral	Bilateral	Left unilateral	Bilateral					The difference between the left unilateral and sham was not significant.	
		Age<60y: 100% RMT, 10Hz, 30s intertrain intervals, 1,450 pulses/session	Age<60y: 100% RMT, R: 1Hz; L: 10Hz, 30s intertrain intervals, R: 465, L: 750 pulses/session	120% AdjRMT, 10Hz, 30s intertrain intervals, 2,100 pulses/session	120% AdjRMT, R:1Hz; L: 10Hz, 30s intertrain intervals, 2,100 pulses/session					There was a modest correction between change in suicidal ideation and change in depression severity.	
		Age>60y: 120% RMT, 10Hz, 30s intertrain intervals, 1,450 pulses/session	Age>60y: 120% RMT, R: 1Hz; L: 10Hz, 30s intertrain intervals, R: 465, L: 750 pulses/session							No difference in change in HDRS‐16 score between suicide remitters and nonremitters.	
Wall et al. (2011)	A prospective, open, multicenter study	120% RMT, 10 Hz, 4‐s train duration, 26‐s intertrain interval, 75 trains, 3,000 pulses/session				30 sessions	5 sessions/w	Baseline, 10th session, 20th sessions, 30th sessions, 6th month	CDRS‐R, 17QIDS‐A, CGI‐S, CGI‐I, C‐SSRS, SRQ, AEMF	Expression of suicidal ideation decreased as treatment progressed, commensurate with mood improvement.	Level 2
Hadley et al. (2011)	A prospective study	120% RMT, 10 Hz, 5‐s train duration, 10‐s intertrain intervals, 6,800 pulses/session				10 sessions	5 sessions/w	Baseline, after each session	BDI, SSI	Aggressive rTMS might be able to significantly and rapidly reduce suicidal ideation.	Level 2
Desmyter et al. (2016)	RCT	110% RMT, 50 Hz, 54 trains of 10 bursts of 3 stimuli/session, repeated every 200ms, 1,620 pulses/session				20 sessions	5 sessions/d	Baseline, 1st w, 2nd w, 1st m, 6th m	SSI, HDRS	A significant decrease in suicide risk was unrelated to active or sham stimulation and unrelated to depression response, lasting up to 1 month.	Level 1a
George et al. (2014)	RCT	120% RMT, 10 Hz, 5‐s train duration, 10‐s intertrain intervals for 30 min, 6,000 pulses/session				9 sessions	3 sessions/d	Baseline, before, and after each rTMS session	SSI, Subjective visual analog scale	A rapid antisuicidal effect by delivering high doses of left prefrontal rTMS over 3 days wasn't demonstrated.	Level 1a
Desmyter et al. (2014)	RCT	100% RMT, 2‐s train duration, 8‐s intertrain interval, 1,620 pulses/session				20 sessions	5 sessions/d	Baseline, 1st w, 2nd w	17‐HDRS, SSI	A significant decrease in SSI score over time was found; unrelated to active or sham stimulation.	Level 1a
Baeken et al. (2019)	RCT	110% RMT, 2‐s train duration, 8‐s intertrain interval, 1,620 pulses/session				20 sessions	5 sessions/d	Baseline, 1st w, 2nd w, 4th w	BDI, SSI	Both active and sham aiTBS resulted in prompt decreases in suicidal ideation. Placebo responses are related to higher cognitive processes resulting in suicidal ideation attenuation.	Level 1a
Croarkin et al. (2018)	A prospective study	120% RMT, 10 Hz, 4‐s train duration, 26‐s intertrain interval, 3,000 pulses/session				30 sessions	3–5 sessions/week	Baseline, after 10 sessions, 20 sessions, 30 sessions	CDRS‐R, C‐SSRS, CGI‐S	The predicted odds of suicidal ideation significantly decreased over 6 weeks of acute TMS treatment without adjustments for illness (depression) severity. The magnitude of the decrease in the predicted odds of suicidal ideation across 6 weeks of treatment was attenuated nonsignificantly in subsequent analyses that adjusted for illness (depression) severity.	Level 2
Berlim et al. (2014)	A prospective study	100% RMT (1er w), gradually increased to 120% during the 2nd w, 20 Hz, 2‐s train duration, 20‐s intertrain interval, 3,000 pulses/session				20 sessions	5 sessions/w	Baseline, 5th w	21‐HDRS, 16‐QIDS‐SR, HAMA, BAI, CGI‐S, SSI	Suicidality ratings were significantly improved by week 5.	Level 2

Abbreviations: Adj RMT, resting motor threshold adjusted for distance; AEMF, Adverse Event Monitoring Form; BAI, Beck Anxiety Inventory; BDI, Beck Depression Inventory; CDRS‐R, Children's Depression Rating Scale‐Revised; CGI‐I, Clinician Global Impressions‐Improvement; CGI‐S, Clinician‐rated global illness severity; C‐SSRS, Columbia Suicide Severity Rating Scale; DTMS, deep transcranial magnetic stimulation; HAMA, Hamilton Anxiety Rating Scale; HDRS, Hamilton depression scale; 17‐HDRS, 17‐item Hamilton depression scale; MADRS, Montgomery–Asberg Depression Rating Scale; RCT, randomized controlled trial; RMT, resting motor threshold; rTMS, repetitive transcranial magnetic stimulation; SRQ, Subjective Reaction Questionnaire; SSI, Beck Scale of Suicidal Ideation; SSI‐CV, Beck Scale for Suicide Ideation‐Chinese Version; 17‐QIDS‐A, 17‐item Quick Inventory of Depressive Symptoms‐Adolescent version; 16‐QIDS‐SR, 16‐item Quick Inventory of Depressive Symptomatology—Self‐Report.

As outlined in Table 5, the only available tDCS study used a current density of 0.8 A/m^2^ (2 mA/25 cm^2^) per 30 min/d using a standard commercial device (Chattanooga Ionto™ Dual Channel Devices, Chattanooga Group). The anode and the cathode were placed over the scalp areas corresponding, respectively, to the left and right DLPFC. tDCS was delivered 5 sessions per week for 2 weeks.

**TABLE 5:** Summary of tDCS study characteristics

Study	Study design	Stimulus dose administration	Number of sessions	Frequency of treatment	Analysis	Primary Outcome	Results/Authors' conclusions	Evidence (PEDro)
Brunoni, Júnior, et al. (2013) and Brunoni, Valiengo, et al. (2013)	RCT	0.8 A/m^2^ (2 mA/25 cm^2^) per 30 min/d	10 sessions	1 session/d	Baseline, 6th w	MADRS	tDCS (alone and combined with sertraline) improved suicidal thoughts.	Level 1a

Abbreviations: MADRS, Montgomery–Asberg Depression Rating Scale; RCT, randomized controlled trial; tDCS, transcranial direct current stimulation.

#### 3.3.3 Associated therapies

Potential add‐on treatments such as medication that can be delivered during the stimulation protocol were not specified in twelve studies (Berlim et al., 2014; George et al., 2014; Hadley et al., 2011; Hunt et al., 2011; Keshtkar et al., 2011; Liang et al., 2017; Munk‐Olsen et al., 2007; Nordenskjöld et al., 2013; Popiolek et al., 2018; Sun et al., 2016, 2018; Weissman et al., 2018). In the studies from Baeken et al. (2019) and Desmyter et al. (2014, 2016) and, rTMS was given as a monotherapy. In other seven studies, participants were demanded to use the same type psychotropic medication to eliminate its medical effects on the outcomes. For instance, ECT was delivered together with antidepressant therapy as an experimental group in two studies (Brådvik & Berglund, 2006; Kellner et al., 2005). More precisely, participants in Wall et al. (2011)’s study only used selective serotonin reuptake inhibitor (SSRI) as an antidepressant. Only Brunoni, Júnior, et al. (2013) and Brunoni, Valiengo, et al. 2013)’s RCT used a fixed antidepressant dosage of 50mg/d of sertraline. In other studies, the medications were finally analyzed and compared with the stimulation group. For instance, Patel et al. (2006) designed a control group with psychotropic pharmacotherapy matched for age, sex, race, and diagnoses, while Ahmadi et al. (2015) included an antidepressant monotherapy group.

#### 3.3.4 Sham stimulation

No sham‐stimulation groups were included in the ECT or MST studies. Five rTMS studies (Baeken et al., 2019; Desmyter et al., 2014, 2016; George et al., 2014; Weissman et al., 2018) and the tDCS study (Brunoni, Júnior, et al., 2013; Brunoni, Valiengo, et al., 2013) were double‐blind sham‐controlled whereby both patients and investigators were blinded to the treatment allocations. In rTMS studies, the sham stimulation was administrated with the coil angled 90° away from the scalp in a single‐wing tilt position producing some cutaneous sensation and similar sound intensity to that of active stimulation (Blumberger et al., 2011, 2016), or a specially designed commercial sham coil, exactly the same as the active coil in appearance, was placed on exactly the same target area of the scalp but without any active stimulation (Baeken et al., 2019; Desmyter et al., 2014, 2016; George et al., 2014). For the tDCS experiment, the sham method consisted of a brief (<30–60 s) period of active stimulation to mimic skin side effects such as tingling and itching before the simulated procedure.

#### 3.3.5 Duration

In the ECT experimental studies, the treatment lasted from 2 weeks (four sessions) for the shortest (Veltman et al., 2018) to 4 months (thirteen sessions) (Kellner et al., 2005) for the longest. The two MST studies did not indicate the accurate duration of experiment. In the rTMS studies, the duration varied from 3 days to 8 weeks and stimulation was delivered one session per day during 10 days in the tDCS protocol. Apart from four ECT studies (Hunt et al., 2011; Liang et al., 2017; Munk‐Olsen et al., 2007; Patel et al., 2006) and two rTMS studies (Berlim et al., 2014; Croarkin et al., 2018), the other eight ECT studies, seven rTMS studies, and the only available tDCS study evaluated participants during follow‐up ranging from weeks to years.

#### 3.3.6 Standardized evaluation

Seventeen studies focused on suicidal ideation as primary outcome for suicidality (Baeken et al., 2019; Berlim et al., 2014; Brunoni, Júnior, et al., 2013; Brunoni, Valiengo, et al., 2013; Ciapparelli et al., 2001; Croarkin et al., 2018; Desmyter et al., 2014; Desmyter et al., 2016; George et al., 2014; Hadley et al., 2011; Kellner et al., 2005; Keshtkar et al., 2011; Patel et al., 2006; Sun et al., 2016, 2018; Veltman et al., 2018; Wall et al., 2011; Weissman et al., 2018), while two studies assessed suicide attempt (Avery & Winokur, 1978; Brådvik & Berglund, 2006) and five studies analyzed rate of completed suicide (Ahmadi et al., 2015; Black et al., 1989; Hunt et al., 2011; Liang et al., 2017; Munk‐Olsen et al., 2007). Popiolek et al. (2018) evaluated suicide attempts and completed suicide at the same time. Nordenskjöld et al. (2013) simply calculated the amount of suspected suicide and suicide attempts.

Different standardized clinical scales to measure suicidal ideation were used across the studies. The Beck Scale of Suicidal Ideation (SSI) was applied in eight studies (Baeken et al., 2019; Berlim et al., 2014; Desmyter et al., 2014, 2016; George et al., 2014; Hadley et al., 2011; Sun et al., 2016, 2018). To assess the severity of suicide, and Croarkin et al. (2018) and Wall et al. (2011) used the Columbia Suicide Severity Rating Scale (C‐SSRS). The third item of the Hamilton depression rating scale (HDRS) or the tenth item of the Montgomery–Asberg Depression Rating Scale (MADRS) were in studies to assess severity of suicidal ideation (Brunoni, Júnior, et al., 2013; Brunoni, Valiengo, et al., 2013; Ciapparelli et al., 2001; Keshtkar et al., 2011; Kellner et al., 2005; Veltman et al., 2018; Weissman et al., 2018). The nine ECT studies did not use any specific scale to measure suicidality but analyzed the occurrence of suicide attempts (Avery & Winokur, 1978; Brådvik & Berglund, 2006; Nordenskjöld et al., 2013; Popiolek et al., 2018), the severity of suicide attempt (Brådvik & Berglund, 2006), suicide events (Liang et al., 2017), and suicide rates (Ahmadi et al., 2015; Black et al., 1989; Hunt et al., 2011; Munk‐Olsen et al., 2007; Popiolek et al., 2018).

A large number of studies also evaluated the severity of depression with various standardized scales: the 17 or 21‐item HDRS (Berlim et al., 2014; Desmyter et al., 2014; Kellner et al., 2005; Weissman et al., 2018), the Beck Depression Inventory (BDI) (Baeken et al., 2019; Hadley et al., 2011; Keshtkar et al., 2011), the 10‐item MADRS (Brunoni, Júnior, et al., 2013; Brunoni, Valiengo, et al., 2013; Ciapparelli et al., 2001; Nordenskjöld et al., 2013; Veltman et al., 2018), and the Children's Depression Rating Scale‐Revised (CDRS‐R) (Croarkin et al., 2018; Wall et al., 2011). The 17‐item Quick Inventory of Depressive Symptoms‐Adolescent version (QIDS‐A17) and the 16‐item Quick Inventory of Depressive Symptomatology‐Self‐Report (16‐QIDS‐SR) were used as the combination of several of these scales or specific scale for adolescents (Berlim et al., 2014; Wall et al., 2011).

### 3.4 Results on suicidality

#### 3.4.1 Results of ECT studies

Ten out of the fourteen ECT studies reported significant efficacy of ECT on suicidality. Five studies highlighted the beneficial effect of ECT on suicidal ideation (Ciapparelli et al., 2001; Kellner et al., 2005; Keshtkar et al., 2011; Patel et al., 2006; Veltman et al., 2018). Another retrospective study observed that ECT can reduce the occurrence of suicide attempts (Brådvik & Berglund, 2006), and suicide attempts were seen significantly less in the ECT group than in the pharmacotherapy‐alone group (Avery & Winokur, 1978; Nordenskjöld et al., 2013). Two other papers reported the long‐term effect of ECT on completed suicides (Ahmadi et al., 2015; Liang et al., 2017). Three ECT studies did not observe any beneficial effects of ECT on suicidality (Black et al., 1989; Hunt et al., 2011; Popiolek et al., 2018). The ECT study published by Munk‐Olsen et al. (2007) described an increase in suicide rates in patients receiving ECT. They believed that the bias (confounding by indication) is a likely explanation for the moderately increased suicide risk among ECT patients in this study and the more marked increase shortly after treatment.

##### Suicidal ideation

Five studies reported the acute effects of ECT on suicidal ideation after acute treatment within 3 weeks (Kellner et al., 2005; Patel et al., 2006), within 1 week after two sessions (Veltman et al., 2018), and one month after approximately nine sessions (Ciapparelli et al., 2001), by evaluating, respectively, the suicide subscale of MADRS (Ciapparelli et al., 2001), and the 24‐item Brief Psychiatric Rating Scale (24‐BPRS) (Patel et al., 2006; Veltman et al., 2018). Within them, Kellner et al. (2005) randomly assigned participants whose condition remitted and remained remitted for one week without treatment, after an acute course of three bilateral ECT sessions for a 6‐month continuative phase, to receive either continual ECT or continual nortriptyline+lithium treatment. The results showed that 38.2% of the patients had a score decrease from 3 or 4 to 0 at item 3 of the 24‐HDRS after 1 week (3 ECT sessions), 61.1% after 2 weeks (6 ECT sessions), and 80.9% at the end of the course of treatment (9 ECT sessions). In addition, comparing the rapid antisuicidal effects with other NIBS like rTMS, Keshtkar et al. (2011) randomized 73 patients with MDD in two groups: one rTMS and one ECT group. After ten sessions, ECT led to greater depressive symptom reductions and suicidal behavior than ten sessions of rTMS, as evaluated by the BDI and HDRS.

##### Suicide attempt

Brådvik & Berglund (2006) collected 96 suicidal and 96 matched controls who were diagnosed as melancholia in the 1950s and 1960s; they principally analyzed the occurrence and severity of suicide attempts following up until 1998. By distinguishing 3 groups: 1) ECT only, 2) antidepressant pharmacotherapy group, and 3) ECT with continuous antidepressant treatment, they demonstrated a better effect of ECT than antidepressants on the occurrence of suicide attempts in a treatment course of at least six sessions (2 weeks). However, on the other hand, they found a worsening on the severity of suicide attempts. Nordenskjöld et al. (2013) saw one suspected suicide and three suicide attempts by intoxication occurred, all in the pharmacotherapy‐alone group but none in ECT plus pharmacotherapy group, while Avery & Winokur (1978) indicated suicide attempts were seen significantly less frequently in the ECT groups (0.8%) than in the antidepressant group (4.2%) or the "adequate" antidepressant subgroup (7.0%).

##### Completed suicide

Liang et al. (2017) compared the cumulative incidence of suicide between ECT (*n* = 487) and non‐ECT recipients (*n* = 1,948) over 13 years. They reported a superior antisuicidal effect of ECT in patients with unipolar disorder and bipolar depression with the adjusted HR present 0.79 (*p* =.041) and 0.805 (*p* =.046) exerted than non‐ECT group. Another retrospective study with the median of 8 years of follow‐up analyzed the death rate, suicide rate, and the relative suicide risk of 92 patients treated with ECT and 3,393 treated with antidepressant monotherapy. Both groups were diagnosed with both PTSD and MDD. The suicide rate was 2.2% and 5.9% in groups with and without ECT, respectively (Ahmadi et al., 2015).

There were some studies that failed to demonstrate that ECT had a preventative effect on suicide in the long term. According to a national clinical survey in England, Hunt et al. (2011) concluded that the reduction in ECT use did not influence suicide rates in patients who received this treatment by collecting an 8‐year sample of suicide cases (1999–2006). Black et al. (1989) included from a short‐term follow‐up study of depressed individuals whom had received ECT in the hospital had minimal influence on suicide. And Popiolek et al. (2018) concluded by their 3‐year follow‐up study that patients treated with continuative ECT had a similar risk of suicide attempts or completed suicides to those who did not receive continuation ECT. Another striking findings by a cohort study demonstrated that 783 inpatients who received ECT had a slightly higher suicide rate (RR = 1.20) compared with 5,871 who had not, especially within the first 7 days from last ECT treatment (Munk‐Olsen et al., 2007).

#### 3.4.2 Results of MST studies

Sun et al. (2016) and (2018) applied the same stimulation MST protocol and assessed the SSI to evaluate the suicidal ideation after 24 MST sessions, or until remission of depressive symptoms. Both trials reported significant reduction in suicidal ideation.

#### 3.4.3 Results of rTMS studies

Statistically significant effects of active rTMS on suicidal ideation were reported in four different studies (Berlim et al., 2014; Hadley et al., 2011; Wall et al., 2011; Weissman et al., 2018). Five studies revealed a beneficial, but nonsignificant effect, of active rTMS on suicidality over sham (Baeken et al., 2019; Croarkin et al., 2018; Desmyter et al., 2014, 2016; George et al., 2014).

Two studies from the same team highlighted the efficacy of left unilateral (10Hz) or bilateral (left: 1Hz; right: 10Hz) DLPFC rTMS on reducing depression in patients with TRD (Blumberger et al., 2011, 2016). Two years later, they integrated data from these two trials and determined that bilateral rTMS was superior to sham rTMS in reducing suicidal ideation in patients with TRD with a significant odds ratio (OR) = 3.03 after fifteen sessions over 3 weeks (Weissman et al., 2018). Another trial analyzing the evidence from nineteen adults with MDD explained that high‐dose left DLPFC (L‐DLPFC) rTMS of 120% RMT and 10 Hz might be a tolerated therapy to rapidly diminish suicidal ideation, as the suicidal ideation of 67% of patients diminished after only five sessions over 1 week (Hadley et al., 2011). Wall et al. (2011) also showed that suicidal ideation progressively decreased during thirty sessions of L‐DLPFC high‐frequency rTMS (10 Hz) in adolescents with TRD by using the same intensity of 120% RMT. Berlim et al (Berlim et al., 2014) applied 5 weeks of daily 20 Hz deep TMS in an open‐labeled fashion in 17 depressed patients, showing improvement in suicidality ratings.

Similar to the results observed with ECT, inconsistencies in rTMS studies were also reported. Despite some studies reporting beneficial effects, some others failed to report any superiority of active rTMS over sham (Baeken et al., 2019; Croarkin et al., 2018; Desmyter et al., 2014; George et al., 2014).

#### 3.4.4 Results of tDCS study

This single tDCS RCT showed significant decreases in suicidal ideation. A significant decrease in suicidal thoughts measured by MADRS 10th item was observed after 10 daily tDCS sessions over sham (*p* = .04) (Brunoni, Júnior, et al., 2013; Brunoni, Valiengo, et al., 2013). This study suggests that patients with depression and suicidal ideation could benefit from tDCS.

## 4 DISCUSSION

In the present review, we have examined the current status of ECT, MST, rTMS, and tDCS in the treatment of suicidal ideation, suicide attempts, and completed suicides.

### 4.1 ECT studies

Among the fourteen retrieved studies, five ECT experimental studies for MDD reported that patients who had received ECT showed significant faster and larger effects on suicidality than control patients groups (pharmacotherapy (Avery & Winokur, 1978; Nordenskjöld et al., 2013; Kellner et al., 2005) or rTMS (Keshtkar et al., 2011) or compared with baseline (Veltman et al., 2018). These findings are consistent with the recommendations of the Canadian Network for Mood and Anxiety Treatments (CANMAT) 2016 Clinical Guidelines for the Management of Adults with Major Depressive Disorder, whereby ECT considered a first‐line treatment for acute suicidal ideation (Milev et al., 2016). A review (Sharma, 2001) of the role of ECT in suicide prevention also recommended the consideration of ECT in treatment algorithms to rapidly reduce suicide rates. The outcomes of these studies seem to be encouraging, and their level of evidence was relatively high (superior to PEDro level 2).

The eight other ECT studies are retrospective or observational studies investigating the occurrence of suicidal behavior in cohorts of patients who received ECT. With a PEDro level 3, their conclusions were less convincing than those from two RCTs and two prospective trials. Among them, three studies did not observe any influence of ECT on completed suicides and suicide attempts (Black et al., 1989; Hunt et al., 2011; Popiolek et al., 2018). The five other open studies showed a positive and significant antisuicidal effect of ECT. Nevertheless, neither Bergfeld et al. (2018) or Hunt et al. (2011) gave valuable explanations or assumptions to explain discrepancies observed between studies regarding the long‐term effect of ECT in their meta‐analyses. Difference may be due to the assumption that there is a relapse in depressive episodes, rather than “incomplete” recovery and psychomotor retardation after ECT, despite an expected good short‐term effect on these symptoms (Brådvik & Berglund, 2004). Sharma, 2001 (Sharma, 2001) suggested that ECT had an acute, but not a long‐term beneficial effect on suicidality. Unfortunately, only Nordenskjöld et al. (2013) and Popiolek et al. (2018) designed or analyzed the efficacy of ECT as a maintenance approach for suicidality, but their conclusions were inconsonant. The lack of evidence on this aspect prevented the authors to systematically estimate its long‐term influence. Considering these reasons, it was suggested to be very prudent to speculate on these findings given the high heterogeneity on long‐term antisuicidal effects.

It is important to note that all the retrospective ECT studies have not investigated the influence of concomitant medication on suicidality. For instance, benzodiazepine could increase the clinical efficacy of ECT when delivered using bitemporal stimulation (Delamarre et al., 2019). Thus, it could modulate antisuicidal of ECT. Following this trend, possibly the best intervention for suicide should be to gather different approaches to maximize neuronal plasticity. Notwithstanding the limited and discordant evidence base for ECT in mood disorders, ECT remains a valuable therapeutic option with superior to PEDro level 2 to acutely reduce suicidal ideations. Continuative ECT treatment, including maintenance pharmacotherapy or psychotherapy, should be sustained during the long term to prolong the remission phase (Çakir & Çağlar, 2017).

### 4.2 MST studies

The aims of Sun et al. (2016) and (2018) were to explore indicators or neuroplasticity of remission of suicidal ideation following MST. With the similar schedule as ECT, usually two to three times per week, an index course of twelve sessions (Milev et al., 2016), we could assume that these two studies evaluated the suicidal ideation at the baseline, and 2 or 3 months after the intervention. The results on suicidal ideation of participants with TRD were reasonable and logical because of its similar antidepressive effect compared with ECT (Kayser et al., 2011). According to CANMAT, its acute efficacy on MDD was recommended with evidence level of 3 (Milev et al., 2016) but its overall recommendation, especially in suicidality, needs to be further explored.

### 4.3 rTMS studies

As observed with ECT studies, our review revealed inconstancies across rTMS studies. Hence, four study effects with PEDro level over 2 concluded that rTMS may have an antisuicidal effect (Berlim et al., 2014; Hadley et al., 2011; Wall et al., 2011; Weissman et al., 2018) whereas four others (Baeken et al., 2019; Desmyter et al., 2014, 2016; George et al., 2014) did not observe a significantly superior antisuicidal effect of active stimulation over sham stimulation. Moreover, Baeken et al. (2019) concluded that sham‐stimulation responses resulted in a greater effect on cognitive process involved in suicidal ideation than active. With a similar high‐dose accelerated protocol, Desmyter et al. (2016) had a powerful evidence level of 1a. These negative and opposite results pushed us to consider the possible pitfalls. For instance, two studies evaluated differences between active and sham stimulation after 3 days of rTMS (George et al., 2014), or 3 days after the last iTBS (Desmyter et al., 2014). However, iTBS is thought to create more robust neuroplasticity effects that likely become evident later following treatment due to the commended neurophysiology (Chung et al., 2014). The evaluation was likely done too soon to find a significant difference between these two groups. Regrettably, Desmyter et al. (2016) designed a crossover trial to evaluate the difference between these two groups after the first week of stimulation to avoid carryover effects that could be expected during the second week. They applied iTBS as an intervention; however, they failed to estimate its profound influence on suicidal ideation after the intervention. To confirm the placebo response, Baeken et al. (2019) reported a significant frontopolar prefrontal perfusion decrease after 4 days of sham iTBS which was related to the attenuation of suicidal ideation. The assumption was that the placebo effects interfered with the final results, but they would only emerge in terms of the suicidal symptoms, but not the depression. Furthermore, due to a lack of meta‐analysis specifically exploring the antisuicidal effects of rTMS, we have not successfully sought out evidence of higher levels to appraise these findings.

Considering that this intervention was recommended by the U.S. Food and Drug Administration (FDA) for the treatment of resistant depression since 2014, accelerated DLPFC‐rTMS is clinically acceptable and could be one of the therapeutic options in addition to its antidepressant effects. Thus, further research is necessary to extend to more naturalistic conditions, and larger sample to confirm its superior antisuicidal effect over sham.

### 4.4 tDCS study

According to the one available study, tDCS could be a choice to decrease suicidality. Another study found that tDCS combined with sertraline was more effective than tDCS, or sertraline alone, on depression. Following this trend, probably the best treatment for suicide might be to combine different interventions, such as pharmacotherapy, to decrease suicidality of patients with depression (Brunoni, Júnior, et al., 2013; Brunoni, Valiengo, et al., 2013).

### 4.5 Safety

In reference to safety, while both rTMS and tDCS are generally considered safe and acceptable if the appropriate guidelines and recommendations are followed, more research is needed to verify the safety parameters of rTMS and tDCS for the purpose of preventing suicidality. The adverse events of ECT potentially could be prevented. It is possible that the routine application of an ECT checklist could diminish the rates of adverse events.

### 4.6 Limitations

Our aim was to evaluate the efficacy between five physical treatments (ECT, MST, rTMS, tDCS, and CES) on suicidality. Due to the limited number of studies and the high heterogeneity between protocols, we were not able to complete a quantitative synthesis and to undertake a meta‐analysis. The efficacy of seizure therapies and NIBS as a continuous intervention also should be further explored as Nordenskjöld et al. (2013) and Popiolek et al. (2018) were the only two studies discussing the long‐term effect of ECT as continuation treatment. None of the MST, rTMS, or tDCS studies investigated MST and/or rTMS and/or tDCS as a continuous intervention to assess its long‐term influence after the acute course of intervention sessions. With a PEDro level of 3 in eight of them, a lack of control group, weak design, and possible cohort effects were obvious methodological shortcomings. Additionally, another obvious limitation of the present review constitutes selection bias. We restricted our search strategy to full‐text articles in English. For example, a systematic review of Norwegian papers revealed doubt upon the efficacy of ECT in the prevention of suicide. We could not critically evaluate those articles or challenge the reported data. Moreover, as mentioned earlier, we had to exclude six studies whose Sackett Scale was equal or inferior to 4 (Brunoni, Júnior, et al., 2013; Brunoni, Valiengo, et al., 2013; Fligelman et al., 2016; Gambill & McLean, 1983; Kobeissi et al., 2011; Pan et al., 2018; Rapinesi et al., 2014;). These case series or uncontrolled pre–post‐tests should also be recognized because of their values. Thirdly, impulsivity, anxiety, and irritability could often drive someone pondering suicide into action. Only George et al. reported greater improvements in anxiety and irritability with sham than with active TMS (George et al., 2014). These symptoms were lack of further evaluations and discussions as related phenotypes.

## 5 CONCLUSION

Taking into consideration all the current evidence, we support the effect of ECT for acute suicidal ideation, but we cannot recommend the same regarding MST, rTMS, or tDCS. However, further research is necessary to determine whether there is any clear and persuadable evidence for the long‐term efficacy of ECT on suicidality and to investigate the potentiation of ECT‐elicited neuroplasticity changes targeting suicide theory. The antisuicidal effect of rTMS with a standard protocol needs to be confirmed. There is also a need for high‐quality tDCS and CES trials designed for suicide. Combination therapy, both in short term and long term, could be a promising asset toward a faster and more sustained amelioration of suicide.

## CONFLICT OF INTEREST

The authors have no conflicts of interest to declare. All authors contributed to and have approved the final submitted version of the manuscript.

## AUTHOR CONTRIBUTION

Authors YC and CM were involved in the conception, design of the review, and screened the articles; Author YC analyzed the data and drafted the manuscript, figures, or tables under the guidance of CM and EP. Authors CM, EP, EL, and JB provided writing assistance and proof reading the article. All authors contributed to and have approved the final submitted version of the manuscript.

## ETHICAL APPROVAL

All analyses were based on previous published studies; therefore, no ethical approval and patient consent are required.

### PEER REVIEW

The peer review history for this article is available at https://publons.com/publon/10.1002/brb3.2144.

## Data Availability

All data generated or analyzed for supporting the findings of this study are available from the corresponding author upon reasonable request.

## References

[brb32144-bib-0001] Ahmadi, N. , Moss, L. , Simon, E. , Nemeroff, C. B. , & Atre‐Vaidya, N. (2015). Efficacy and long‐term clinical outcome of comorbid posttraumatic stress disorder and major depressive disorder after electroconvulsive therapy. Depression and Anxiety, 33(7), 640–647. 10.1002/da.22451 26555786

[brb32144-bib-0002] Andrade, C. (2017). Ketamine for depression, 1: Clinical summary of issues related to efficacy, adverse effects, and mechanism of action. Journal of Clinical Psychiatry, 78(4), e415–e419. 10.4088/JCP.17f11567 28448702

[brb32144-bib-0003] Avery, D. , & Winokur, G. (1978). Suicide, attempted suicide, and relapse rates in depression. Archives of General Psychiatry, 35(6), 749–753. 10.1001/archpsyc.1978.01770300091010 655772

[brb32144-bib-0004] Baeken, C. , Wu, G.‐R. , & van Heeringen, K. (2019). Placebo aiTBS attenuates suicidal ideation and frontopolar cortical perfusion in major depression. Translational Psychiatry, 9(1), 38. 10.1038/s41398-019-0377-x 30696807 PMC6351528

[brb32144-bib-0005] Bergfeld, I. O. , Mantione, M. , Figee, M. , Schuurman, P. R. , Lok, A. , & Denys, D. (2018). Treatment‐resistant depression and suicidality. Journal of Affective Disorders, 10.1016/j.jad.2018.04.016 29665520

[brb32144-bib-0006] Berlim, M. T. , Van den Eynde, F. , Tovar‐Perdomo, S. , Chachamovich, E. , Zangen, A. , & Turecki, G. (2014). Augmenting antidepressants with deep transcranial magnetic stimulation (DTMS) in treatment‐resistant major depression. The World Journal of Biological Psychiatry, 15(7), 570–578. 10.3109/15622975.2014.925141 25050453

[brb32144-bib-0007] Black, D. , Winokur, G. , Mohandoss, E. , Woolson, R. , & Nasrallah, A. (1989). Does Treatment Influence Mortality in Depressives?: A Follow‐up of 1076 Patients with Major Affective Disorders. Annals of Clinical Psychiatry, 1(3), 165–173. 10.3109/10401238909149975

[brb32144-bib-0008] Blumberger, D. M. , Maller, J. J. , Thomson, L. , Mulsant, B. H. , Rajji, T. K. , Maher, M. , Brown, P. E. , Downar, J. , Vila‐Rodriguez, F. , Fitzgerald, P. B. , & Daskalakis, Z. J. (2016). Unilateral and bilateral MRI‐targeted repetitive transcranial magnetic stimulation for treatment‐resistant depression: A randomized controlled study. Journal of Psychiatry & Neuroscience, 41(4), E58–E66. 10.1503/jpn.150265 27269205 PMC4915938

[brb32144-bib-0009] Blumberger, D. M. , Mulsant, B. H. , Fitzgerald, P. B. , Rajji, T. K. , Ravindran, A. V. , Young, L. T. , Levinson, A. J. , & Daskalakis, Z. J. (2011). A randomized double‐blind sham‐controlled comparison of unilateral and bilateral repetitive transcranial magnetic stimulation for treatment‐resistant major depression. The World Journal of Biological Psychiatry, 13(6), 423–435. 10.3109/15622975.2011.579163 21736507

[brb32144-bib-0010] Brådvik, L. , & Berglund, M. (2004). Suicide in severe depression related to treatment. European Archives of Psychiatry and Clinical Neuroscience, 255(4), 245–250. 10.1007/s00406-004-0553-7 16133742

[brb32144-bib-0011] Brådvik, L. , & Berglund, M. (2006). Long‐term treatment and suicidal behavior in severe depression: ECT and antidepressant pharmacotherapy may have different effects on the occurrence and seriousness of suicide attempts. Depression and Anxiety, 23(1), 34–41. 10.1002/da.20134 16315268

[brb32144-bib-0012] Brunoni, A. R. , Júnior, R. F. , Kemp, A. H. , Lotufo, P. A. , Benseñor, I. M. , & Fregni, F. (2013). Differential improvement in depressive symptoms for tDCS alone and combined with pharmacotherapy: An exploratory analysis from The Sertraline Vs. Electrical Current Therapy For Treating Depression Clinical Study. The International Journal of Neuropsychopharmacology, 17(01), 53–61. 10.1017/S1461145713001065 24060107

[brb32144-bib-0013] Brunoni, A. R. , Valiengo, L. , Baccaro, A. , Zanão, T. A. , de Oliveira, J. F. , Goulart, A. , Boggio, P. S. , Lotufo, P. A. , Benseñor, I. M. , & Fregni, F. (2013). The sertraline vs electrical current therapy for treating depression clinical study. JAMA Psychiatry, 70(4), 383. 10.1001/2013.jamapsychiatry.32 23389323

[brb32144-bib-0014] Çakir, S. , & Çağlar, N. (2017). Electroconvulsive therapy in the treatment of mood disorders: One‐year follow‐up. Noro Psikiyatri Arsivi, 54(3), 196–201. 10.5152/npa.2016.14845 29033629 PMC5630095

[brb32144-bib-0015] Cheng, A. T. A. , Chen, T. H. H. , Chen, C.‐C. , & Jenkins, R. (2000). Psychosocial and psychiatric risk factors for suicide: Case‐control psychological autopsy study. British Journal of Psychiatry, 177(04), 360–365. 10.1192/bjp.177.4.360 11116779

[brb32144-bib-0016] Chung, S. W. , Hoy, K. E. , & Fitzgerald, P. B. (2014). Theta‐burst stimulation: A new form of TMS treatment for depression? Depression and Anxiety, 32(3), 182–192. 10.1002/da.22335 25450537

[brb32144-bib-0017] Ciapparelli, A. , Dell’Osso, L. , Tundo, A. , Pini, S. , Chiavacci, M. C. , Di Sacco, I. , & Cassano, G. B. (2001). Electroconvulsive therapy in medication‐nonresponsive patients with mixed mania and bipolar depression. Journal of Clinical Psychiatry, 62(7), 552–555. 10.4088/JCP.v62n07a09 11488367

[brb32144-bib-0018] Croarkin, P. E. , Nakonezny, P. A. , Deng, Z.‐D. , Romanowicz, M. , Voort, J. L. V. , Camsari, D. D. , Schak, K. M. , Port, J. D. , & Lewis, C. P. (2018). High‐frequency repetitive TMS for suicidal ideation in adolescents with depression. Journal of Affective Disorders, 239, 282–290. 10.1016/j.jad.2018.06.048 30031247 PMC6431788

[brb32144-bib-0019] DaSilva, A. F. , Volz, M. S. , Bikson, M. , & Fregni, F. (2011). Electrode positioning and montage in transcranial direct current stimulation. Journal of Visualized Experiments, (51), 1–11. 10.3791/2744 PMC3339846 21654618

[brb32144-bib-0020] DeCou, C. R. , Comtois, K. A. , & Landes, S. J. (2018). Dialectical behavior therapy is effective for the treatment of suicidal behavior: A meta‐analysis. Behavior Therapy, 50(1), 60–72. 10.1016/j.beth.2018.03.009 30661567

[brb32144-bib-0021] Delamarre, L. , Galvao, F. , Gohier, B. , Poulet, E. , & Brunelin, J. (2019). How much do benzodiazepines matter for electroconvulsive therapy in patients with major depression? The Journal of ECT, 35(3), 184–188. 10.1097/YCT.0000000000000574 30720551

[brb32144-bib-0022] Desmyter, S. , Duprat, R. , Baeken, C. , Bijttebier, S. , & van Heeringen, K. (2014). The acute effects of accelerated repetitive Transcranial Magnetic Stimulation on suicide risk in unipolar depression: Preliminary results. Psychiatria Danubina, 1, 48–52. 25413512

[brb32144-bib-0023] Desmyter, S. , Duprat, R. , Baeken, C. , Van Autreve, S. , Audenaert, K. , & van Heeringen, K. (2016). Accelerated intermittent theta burst stimulation for suicide risk in therapy‐resistant depressed patients: A randomized, sham‐controlled trial. Frontiers in Human Neuroscience, 10, 480. 10.3389/fnhum.2016.00480 27729854 PMC5037167

[brb32144-bib-0024] Desmyter, S. , van Heeringen, C. , & Audenaert, K. (2011). Structural and functional neuroimaging studies of the suicidal brain. Progress in Neuro‐Psychopharmacology and Biological Psychiatry, 35(4), 796–808. 10.1016/j.pnpbp.2010.12.026 21216267

[brb32144-bib-0025] Fligelman, B. , Pham, T. , Bryson, E. O. , Majeske, M. , & Kellner, C. H. (2016). Resolution of acute suicidality after a single right unilateral electroconvulsive therapy. The Journal of ECT, 32(1), 71–72. 10.1097/YCT.0000000000000258 26110919

[brb32144-bib-0026] Gambill, J. D. , & McLean, P. E. (1983). B. Suicide after unilateral ect in a patient previously responsive to bilateral ect. Psychiatric Quarterly, 55(4), 279–281. 10.1007/BF01074556 6680195

[brb32144-bib-0027] George, M. S. , Raman, R. , Benedek, D. M. , Pelic, C. G. , Grammer, G. G. , Stokes, K. T. , Schmidt, M. , Spiegel, C. , DeAlmeida, N. , Beaver, K. L. , Borckardt, J. J. , Sun, X. , Jain, S. , & Stein, M. B. (2014). A two‐site pilot randomized 3 day trial of high dose left prefrontal repetitive transcranial magnetic stimulation (rTMS) for suicidal inpatients. Brain Stimulation, 7(3), 421–431. 10.1016/j.brs.2014.03.006 24731434

[brb32144-bib-0028] Guyatt, G. H. , Oxman, A. D. , Sultan, S. , Glasziou, P. , Akl, E. A. , Alonso‐Coello, P. , Atkins, D. , Kunz, R. , Brozek, J. , Montori, V. , Jaeschke, R. , Rind, D. , Dahm, P. , Meerpohl, J. , Vist, G. , Berliner, E. , Norris, S. , Falck‐Ytter, Y. , Murad, M. H. , & Schünemann, H. J. (2011). GRADE guidelines: 9. Rating up the quality of evidence. Journal of Clinical Epidemiology, 64(12), 1311–1316. 10.1016/j.jclinepi.2011.06.004 21802902

[brb32144-bib-0029] Hadley, D. , Anderson, B. S. , Borckardt, J. J. , Arana, A. , Li, X. , Nahas, Z. , & George, M. S. (2011). Safety, tolerability, and effectiveness of high doses of adjunctive daily left prefrontal repetitive transcranial magnetic stimulation for treatment‐resistant depression in a clinical setting. The Journal of ECT, 27(1), 18–25. 10.1097/YCT.0b013e3181ce1a8c 21343710

[brb32144-bib-0030] Hunt, I. M. , Windfuhr, K. , Swinson, N. , Shaw, J. , Appleby, L. , & Kapur, N. (2011). Electroconvulsive therapy and suicide among the mentally ill in England: A national clinical survey. Psychiatry Research, 187(1–2), 145–149. 10.1016/j.psychres.2010.12.014 21208662

[brb32144-bib-0031] Kayser, S. , Bewernick, B. H. , Grubert, C. , Hadrysiewicz, B. L. , Axmacher, N. , & Schlaepfer, T. E. (2011). Antidepressant effects, of magnetic seizure therapy and electroconvulsive therapy, in treatment‐resistant depression. Journal of Psychiatric Research, 45(5), 569–576. 10.1016/j.jpsychires.2010.09.008 20951997

[brb32144-bib-0032] Kellner, C. H. , Fink, M. , Knapp, R. , Petrides, G. , Husain, M. , Rummans, T. , Mueller, M. , Bernstein, H. , Rasmussen, K. , O’Connor, K. , Smith, G. , Rush, A. J. , Biggs, M. , McClintock, S. , Bailine, S. , & Malur, C. (2005). Relief of expressed suicidal INTENT by ECT: a consortium for research in ECT study. American Journal of Psychiatry, 162(5), 977–982. 10.1176/appi.ajp.162.5.977 PMC3684568 15863801

[brb32144-bib-0033] Keshtkar, M. , Ghanizadeh, A. , & Firoozabadi, A. (2011). Repetitive transcranial magnetic stimulation versus electroconvulsive therapy for the treatment of major depressive disorder, a randomized controlled clinical trial. The Journal of ECT, 27(4), 310–314. 10.1097/YCT.0b013e318221b31c 22080240

[brb32144-bib-0034] Kobeissi, J. , Aloysi, A. , Tobias, K. , Popeo, D. , & Kellner, C. H. (2011). Resolution of severe suicidality with a single electroconvulsive therapy. The Journal of ECT, 27(1), 86–88. 10.1097/YCT.0b013e3181da842a 21343715

[brb32144-bib-0035] Lefaucheur, J.‐P. , André‐Obadia, N. , Antal, A. , Ayache, S. S. , Baeken, C. , Benninger, D. H. , Cantello, R. M. , Cincotta, M. , de Carvalho, M. , De Ridder, D. , Devanne, H. , Di Lazzaro, V. , Filipović, S. R. , Hummel, F. C. , Jääskeläinen, S. K. , Kimiskidis, V. K. , Koch, G. , Langguth, B. , Nyffeler, T. , … Garcia‐Larrea, L. (2014). Evidence‐based guidelines on the therapeutic use of repetitive transcranial magnetic stimulation (rTMS). Clinical Neurophysiology, 125(11), 2150–2206. 10.1016/j.clinph.2014.05.021 25034472

[brb32144-bib-0036] Lefaucheur, J.‐P. , Antal, A. , Ayache, S. S. , Benninger, D. H. , Brunelin, J. , Cogiamanian, F. , Cotelli, M. , De Ridder, D. , Ferrucci, R. , Langguth, B. , Marangolo, P. , Mylius, V. , Nitsche, M. A. , Padberg, F. , Palm, U. , Poulet, E. , Priori, A. , Rossi, S. , Schecklmann, M. , … Paulus, W. (2017). Evidence‐based guidelines on the therapeutic use of transcranial direct current stimulation (tDCS). Clinical Neurophysiology, 128(1), 56–92. 10.1016/j.clinph.2016.10.087 27866120

[brb32144-bib-0037] Liang, C.‐S. , Chung, C.‐H. , Ho, P.‐S. , Tsai, C.‐K. , & Chien, W.‐C. (2017). Superior anti‐suicidal effects of electroconvulsive therapy in unipolar disorder and bipolar depression. Bipolar Disorders, 20(6), 539–546. 10.1111/bdi.12589 29227012

[brb32144-bib-0038] Liberati, A. , Altman, D. G. , Tetzlaff, J. , Mulrow, C. , Gøtzsche, P. C. , Ioannidis, J. P. A. , Clarke, M. , Devereaux, P. J. , Kleijnen, J. , & Moher, D. (2009). The PRISMA statement for reporting systematic reviews and meta‐analyses of studies that evaluate health care interventions: Explanation and elaboration. Journal of Clinical Epidemiology, 62(10), e1–e34. 10.1016/j.jclinepi.2009.06.006 19631507

[brb32144-bib-0039] Milev, R. V. , Giacobbe, P. , Kennedy, S. H. , Blumberger, D. M. , Daskalakis, Z. J. , Downar, J. , Modirrousta, M. , Patry, S. , Vila‐Rodriguez, F. , Lam, R. W. , MacQueen, G. M. , Parikh, S. V. , & Ravindran, A. V. (2016). Canadian Network for Mood and Anxiety Treatments (CANMAT) 2016 clinical guidelines for the management of adults with major depressive disorder. The Canadian Journal of Psychiatry, 61(9), 561–575. 10.1177/0706743716660033 27486154 PMC4994792

[brb32144-bib-0040] Munk‐Olsen, T. , Laursen, T. M. , Videbech, P. , Mortensen, P. B. , & Rosenberg, R. (2007). All‐cause mortality among recipients of electroconvulsive therapy. British Journal of Psychiatry, 190(05), 435–439. 10.1192/bjp.bp.106.026740 17470959

[brb32144-bib-0041] Nordenskjöld, A. , von Knorring, L. , Ljung, T. , Carlborg, A. , Brus, O. , & Engström, I. (2013). Continuation electroconvulsive therapy with pharmacotherapy versus pharmacotherapy alone for prevention of relapse of depression. The Journal of ECT, 29(2), 86–92. 10.1097/YCT.0b013e318276591f 23303421

[brb32144-bib-0042] Pan, F. , Li, D. , Wang, X. , Lu, S. , Xu, Y. , & Huang, M. (2018). Neuronavigation‐guided high‐dose repetitive transcranial magnetic stimulation for the treatment of depressive adolescents with suicidal ideation: A case series. Neuropsychiatric Disease and Treatment, 14, 2675–2679. 10.2147/ndt.s176125 30349265 PMC6188184

[brb32144-bib-0043] Patel, M. , Patel, S. , Hardy, D. W. , Benzies, B. J. , & Tare, V. (2006). Should electroconvulsive therapy be an early consideration for suicidal patients? The Journal of ECT, 22(2), 113–115. 10.1097/00124509-200606000-00007 16801826

[brb32144-bib-0044] Phillips, M. R. , Yang, G. , Zhang, Y. , Wang, L. , Ji, H. , & Zhou, M. (2002). Risk factors for suicide in China: A national case‐control psychological autopsy study. The Lancet, 360(9347), 1728–1736. 10.1016/S0140-6736(02)11681-3 12480425

[brb32144-bib-0045] Plans, L. , Barrot, C. , Nieto, E. , Rios, J. , Schulze, T. G. , Papiol, S. , Mitjans, M. , Vieta, E. , & Benabarre, A. (2018). Association between completed suicide and bipolar disorder: A systematic review of the literature. Journal of Affective Disorders, 242, 111–122. 10.1016/j.jad.2018.08.054 30173059

[brb32144-bib-0046] Popiolek, K. , Brus, O. , Elvin, T. , Landén, M. , Lundberg, J. , Nordanskog, P. , & Nordenskjöld, A. (2018). Rehospitalization and suicide following electroconvulsive therapy for bipolar depression–A population‐based register study. Journal of Affective Disorders, 226, 146–154. 10.1016/j.jad.2017.09.030 28982047

[brb32144-bib-0047] Rapinesi, C. , Kotzalidis G. D. , Scatena, P. , Del Casale, A. , Janiri, D. , Callovini, G. , Piacentino, D. , Serata, D. , Raccah, R. N. , Brugnoli, R. , & Digiacomantonio, V. (2014). Alcohol and suicidality: Could deep transcranial magnetic stimulation (dTMS) be a possible treatment? Psychiatria Danubina., 26(3), 281–284. 25191777

[brb32144-bib-0048] Rosa, M. A. , & Lisanby, S. H. (2011). Somatic treatments for mood disorders. Neuropsychopharmacology, 37(1), 102–116. 10.1038/npp.2011.225 21976043 PMC3238088

[brb32144-bib-0049] Sharma, V. (2001). The effect of electroconvulsive therapy on suicide risk in patients with mood disorders. The Canadian Journal of Psychiatry, 46(8), 704–709. 10.1177/070674370104600802 11692972

[brb32144-bib-0050] Sun, Y. , Blumberger, D. M. , Mulsant, B. H. , Rajji, T. K. , Fitzgerald, P. B. , Barr, M. S. , Downar, J. , Wong, W. , Farzan, F. , & Daskalakis, Z. J. (2018). Magnetic seizure therapy reduces suicidal ideation and produces neuroplasticity in treatment‐resistant depression. Transl Psychiatry, 8(1). 10.1038/s41398-018-0302-8 PMC6251931 30470735

[brb32144-bib-0051] Sun, Y. , Farzan, F. , Mulsant, B. H. , Rajji, T. K. , Fitzgerald, P. B. , Barr, M. S. , Downar, J. , Wong, W. , Blumberger, D. M. , & Daskalakis, Z. J. (2016). Indicators for remission of suicidal ideation following magnetic seizure therapy in patients with treatment‐resistant depression. JAMA Psychiatry, 73(4), 337. 10.1001/jamapsychiatry.2015.3097 26981889

[brb32144-bib-0053] UK ECT Review Group . (2003). Efficacy and safety of electroconvulsive therapy in depressive disorders: a systematic review and meta‐analysis. The Lancet, 361(9360), 799–808. 10.1016/s0140-6736(03)12705-5 12642045

[brb32144-bib-0054] Veltman, E. M. , van Hulten, S. , Twisk, J. , Dols, A. , van Exel, E. , Stek, M. L. , Sienaert, P. , Bouckaert, F. , van der Mast, R. C. , & Rhebergen, D. (2018). Differences in speed of response of depressive symptom dimensions in older persons during electroconvulsive therapy. The Journal of ECT, 35(1), 35–39. 10.1097/YCT.0000000000000506 29847351

[brb32144-bib-0055] Wall, C. A. , Croarkin, P. E. , Sim, L. A. , Husain, M. M. , Janicak, P. G. , Kozel, F. A. , Emslie, G. J. , Dowd, S. M. , & Sampson, S. M. (2011). Adjunctive use of repetitive transcranial magnetic stimulation in depressed adolescents: A prospective, open pilot study. Journal of Clinical Psychiatry, 72(9), 1263–1269. 10.4088/JCP.11m07003 21951987

[brb32144-bib-0056] Weissman, C. R. , Blumberger, D. M. , Brown, P. E. , Isserles, M. , Rajji, T. K. , & Downar, J. D. (2018). Bilateral repetitive transcranial magnetic stimulation decreases suicidal ideation in depression. The Journal of Clinical Psychiatry, 79(3), 17m11692. 10.4088/JCP.17m11692 29701939

[brb32144-bib-0057] WHO . (2019). Title of subordinate document. In: Suicide. Retrieved from http://www.who.int/news‐room/fact‐sheets/detail/suicide

[brb32144-bib-0058] Zalsman, G. , Hawton, K. , Wasserman, D. , van Heeringen, K. , Arensman, E. , Sarchiapone, M. , Carli, V. , Höschl, C. , Barzilay, R. , Balazs, J. , Purebl, G. , Kahn, J. P. , Sáiz, P. A. , Lipsicas, C. B. , Bobes, J. , Cozman, D. , Hegerl, U. , & Zohar, J. (2016). Suicide prevention strategies revisited: 10‐year systematic review. The Lancet Psychiatry, 3(7), 646–659. 10.1016/S2215-0366(16)30030-X 27289303

